# Non-invasive brain stimulation for the improvement of lower extremity motor function in patients with stroke: a systematic review and network meta-analysis

**DOI:** 10.3389/fneur.2025.1664707

**Published:** 2025-12-01

**Authors:** Enliang Deng, Jiayu Li, Lang Zhang, Xin Zhou, Zhen Wu, Wuhua Xu, Dongmei Jin

**Affiliations:** 1 Guangzhou Red Cross Hospital, Guangzhou, China; 2 Oncology Department, Shantou Central Hospital, Shantou, China; 3 967 Hospital of the Joint Logistics Support Force, Dalian, Liaoning, China; 4 The Second Medical Center, Chinese PLA General Hospital, Beijing, China; 5 Sun Yat-Sen Memorial Hospital, Sun Yat-sen University, Guangzhou, China

**Keywords:** rTMS (repetitive transcranial magnetic stimulation), tDCS, stroke—diagnosis, lower limb and rehabilitation, NIBS (non-invasive brain stimulation)

## Abstract

**Objective:**

To explore and compare the effectiveness of various non-invasive brain stimulations (NiBS) on poststroke lower extremity disorders.

**Methods:**

We searched for and gathered studies from Embase, PubMed, Web of Science, and Cochrane databases, with the most recent search carried out on 5 October 2024. All published studies meeting the eligibility criteria and investigating the effectiveness of NiBS in patients with poststroke lower limb disorders were included. A total of 29 studies involving 1,319 participants were reviewed. Two independent researchers extracted clinical characteristics and research data. Outcome measures included the Fugl–Meyer lower extremity scale, Barthel index, Berg balance scale (BBS), and timed up and go test. Standard pairwise meta-analysis results and treatment network geometry were generated using Stata MP version 15.0. Bayesian network analysis was conducted using R version 4.4.1 with the “BUGSnet” package.

**Conclusion:**

The meta-analysis shows that low-frequency repetitive transcranial magnetic stimulation (LF-rTMS) and rTMS + transcranial direct current stimulation (tDCS) are effective neurostimulation therapies for enhancing poststroke lower limb motor function. Probability rankings indicate that, among all NiBS interventions examined, rTMS + tDCS may be the most effective. In terms of body balance, intermittent theta burst stimulation (iTBS) and LF-rTMS improved poststroke balance, with iTBS possibly being the most effective. For activities of daily living, iTBS, LF-rTMS, and rTMS + tDCS demonstrated beneficial effects, with LF-rTMS potentially being the most effective among them.

## 1 Introduction

As the population ages, the incidence of stroke continues to rise (1). Lower extremity dysfunction is a common post-stroke functional impairment. This dyskinesia persists for a long time, hindering daily activities, reducing muscle strength, and limiting work-related activities and social participation (2). Current rehabilitation approaches for post-stroke lower limb motor dysfunction mainly include repetitive task-oriented training, walking exercises, treadmill training, orthotics, and functional electrical stimulation (3). However, these traditional therapies are time-consuming and produce inconsistent results. Therefore, developing innovative treatment methods that enhance balance, walking ability, and performance of daily living activities is vital in stroke rehabilitation research.

Non-invasive brain stimulation (NiBS) includes emerging techniques used in neurorehabilitation to restore motor function after stroke by modulating the excitability of motor control centers (4). NiBS techniques include transcranial ultrasound stimulation, transcranial direct current stimulation (tDCS), and transcranial magnetic stimulation (TMS) (5). However, relatively few clinical studies have explored the effectiveness of transcranial ultrasound stimulation for poststroke motor function recovery (6). Based on various stimulation patterns, TMS techniques are classified into single-pulse TMS, dual-pulse TMS, repetitive TMS (rTMS), and the derived rTMS mode (theta burst stimulation, TBS) (7).

A considerable number of clinical studies have been published on treating poststroke lower limb movement disorders using NiBS techniques. These studies utilise different stimulation modes, including low-frequency rTMS (LF-rTMS), high-frequency rTMS (HF-rTMS), combined rTMS and transcranial direct current stimulation (rTMS + tDCS), intermittent TBS (iTBS), continuous TBS (cTBS), anodal tDCS (A-tDCS), dual-tDCS, and cathodal tDCS (C-tDCS). Reported outcomes include the Fugl–Meyer assessment for the lower extremity (FMA-LE), the Barthel index (BI), the Berg balance scale (BBS), and the timed up and go test (TUG) (8, 9). Based on these studies, several meta-analyses have evaluated the effectiveness of various NiBS therapies in treating post-stroke motor disorders (10, 11). Traditional meta-analyses, however, are limited to pairwise comparisons and cannot establish a comprehensive treatment hierarchy (network evidence), as their results are based on direct comparisons of relevant treatments. In contrast, network meta-analysis (NMA) is a relatively new statistical method that combines, compares, and integrates multiple interventions within a single analysis. Although a large number of traditional pairwise comparisons are needed to support such integration, NMA enables ranking of all interventions using both direct trial data and indirect evidence from cross-comparisons (12). To evaluate and compare the effectiveness of various NiBS treatments for lower extremity disorders in post-stroke patients, we conducted a literature search and synthesized the available evidence in this review.

## 2 Methods

The study protocol was registered in PROSPERO (CRD42024521395) on May 20, 2024.^1^ We prepared the NMA following the Preferred Reporting Items for Systematic Review and Meta-analysis Protocols (PRISMA-P) statement (13).

### 2.1 Eligibility criteria

Studies meeting the following criteria were included in the meta-analysis: (1) participants diagnosed with lower limb paralysis after stroke; (2) intervention involving NiBS, including rTMS, tDCS, specialized modes of rTMS, and the combined use of multiple NiBS techniques (no relevant studies identified for other NiBS modalities); (3) comparison using placebo conditions, such as sham stimulation or blank controls; (4) outcomes measured with TUG, FMA-LE, BI, and BBS; and (5) research limited to randomized controlled trials (RCTs).

Studies were excluded for the following reasons: (1) recruiting ineligible participants, such as healthy populations or animals; (2) using unrelated interventions, like invasive deep brain stimulation; (3) having unclear stimulation patterns; (4) when research data was inaccessible or incomplete; (5) being published as meetings, case reports, or reviews; and (6) duplicate publications.

### 2.2 Data sources and searches

We searched for relevant literature in the following databases, with the last search ending on October 5, 2024: PubMed, Embase, the Cochrane Library, and Web of Science. The keywords, including MeSH terms related to the lower extremities, stroke, tDCS, and TMS, are listed in the Supplementary file.

### 2.3 Data collection and analysis

Two independent researchers (DEL and LJY) screened potentially relevant studies based on titles, abstracts, and full texts. In cases of disagreements, a third researcher was consulted to make the final decision. After scanning the included studies, the following information was extracted: publication date, author names, stimulation area, stroke subtype (ischemic/hemorrhagic), time of onset, sex, sample size, age, and adverse effects.

#### 2.3.1 Quality assessment

We used Review Manager (version 5.4), based on the Cochrane risk of bias assessment tool, to assess risk of bias in RCTs across seven domains (14). Two independent researchers (DEL and LJY) assessed the studies according to these domains, which are listed in Supplementary file 2. To determine potential publication bias among the included studies, we applied Egger’s test using Stata MP (version 15). A *p*-value <0.05 was considered to indicate that the results of the meta-analysis were unreliable (15).

#### 2.3.2 Outcomes and effect measures

Four outcomes were used to evaluate the effectiveness of NiBS for poststroke lower extremity movement disorders: FMA-LE, TUG, BI, and BBS. For a thorough assessment of lower extremity motor recovery, the primary outcome was the FMA-LE, a tool commonly used to assess motor function in patients with stroke or other central nervous system diseases. This scale thoroughly evaluates lower limb function, with higher scores indicating better recovery. Secondary outcomes included the TUG, BI, and BBS. The TUG is a quick assessment test that measures walking ability by recording the time needed to complete the test. Shorter times reflect better walking function. The BBS is a detailed scale used to assess body balance function, with higher scores indicating better balance performance. The BI is a widely used tool to evaluate activities of daily living and is mainly useful for detecting changes in independent living abilities of elderly individuals before and after treatment. Higher BI scores suggest better performance in activities of daily living.

For all outcomes treated as continuous variables, we set the mean difference (MD) as the effect size, with a 95% confidence interval (CI). To calculate the effect measures for continuous outcomes, the outcomes before and after NiBS were recorded as means and standard deviations.

#### 2.3.3 Geometry of the network

Network graphs were established to visualize the characteristics of the included NiBS techniques and to compare them with the placebo group. Each node in the network graph represents an NiBS technique. Node size indicates the number of subjects, and the lines between nodes represent random comparisons between intervention measures.

### 2.4 Statistical analysis

#### 2.4.1 Methods for direct treatment comparisons

Based on the results of statistical heterogeneity, we applied a random-effects model to assess the direct relative effects between competing NiBS techniques and the placebo using Stata MP version 15.0.

#### 2.4.2 Methods for indirect and mixed comparisons

Bayesian network analysis, based on the Markov chain Monte Carlo algorithm, was applied to assess the effectiveness of each NiBS therapy by R version 4.4.1 with the “BUGSnet” package. We applied the deviance information criterion (DIC) to guide model selection between fixed- and random-effects approaches, and the model with the lower DIC was chosen to ensure a better fit. All NiBS techniques were ranked according to their P-scores, which ranged from 0 to 1. The results are shown in a surface under the cumulative ranking curve (SUCRA) plot. Comparison results are reported as MD with 95% credible intervals, presented in a league table.

#### 2.4.3 Assessment of statistical heterogeneity and inconsistency

For standard pairwise meta-analysis, we used the *I^2^* statistic to assess statistical heterogeneity, with values over 50% indicating significant heterogeneity. For indirect and mixed comparisons, inconsistencies were assessed at both global and local levels. At the global level, inconsistency was evaluated by calculating the DIC from the inconsistency model and comparing it to the consistency model. A difference of less than 5 between the two models was deemed insufficient to indicate network inconsistency. To assess local inconsistency, leverage plots were created, and the scatter of data points was examined.

## 3 Results

### 3.1 Study selection

**Figure 1:**
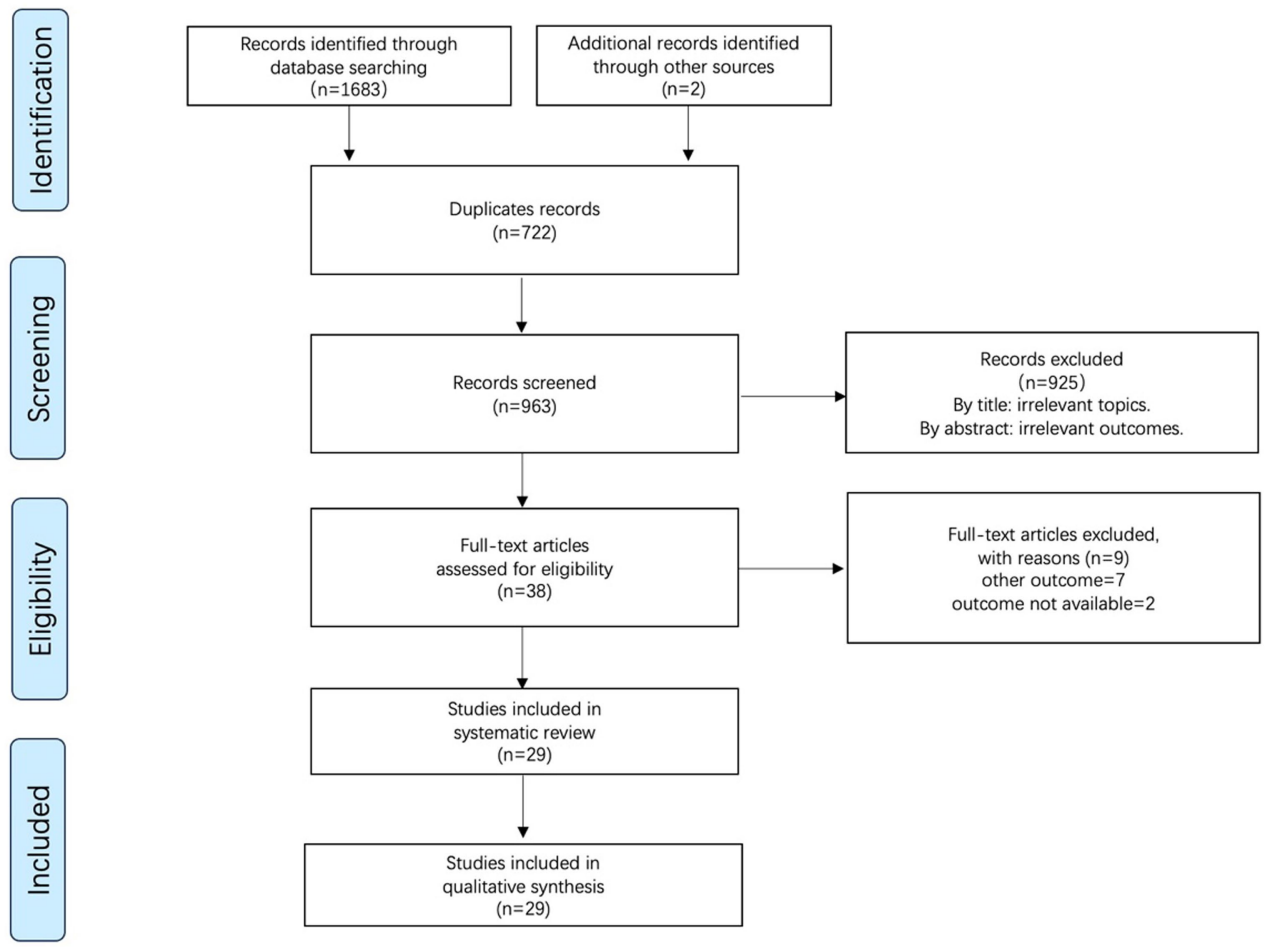
PRISMA flow diagram for study selection.

We collected 1,683 studies from four electronic databases: PubMed (*n* = 415), Embase (*n* = 352), WOS (*n* = 618), and Cochrane (*n* = 298). Additionally, two studies were included after reviewing other reviews. A total of 722 duplicate studies identified using Endnote’s duplicate citation checker were excluded. After reading and screening the titles and abstracts, 925 studies were excluded. Following full-text review of the remaining 38 studies, we excluded nine studies for the following reasons: other outcomes = 7 and unavailable outcome data = 2. Finally, 29 studies were included in the quantitative analysis. The PRISMA flow diagram for study selection is shown in Figure 1.

### 3.2 Study characteristics

A comprehensive summary of the characteristics of the included studies is presented in Table 1. Of the 29 included studies, 28 were RCTs, except for 1 crossover trial (16). For the 29 studies involving 1,319 participants, LF-rTMS was used in 9 studies (8, 9, 17–23), HF-rTMS in 4 studies (18, 24–26), bil-rTMS in 1 study (8), iTBS in 5 studies (7, 27–30), cTBS in 1 study (8), C-tDCS in 1 study (31), A-tDCS in 6 studies (32–37), dual-tDCS in 4 studies (16, 38–40), and rTMS + tDCS in 2 studies (9, 41).

**Table 1:** Summary of the characteristics of included studies.

Study	Intervention	Area of stimulation	Stroke subtype (ischemic/hemorrhagic)	Time of onset (mean ± SD)	Sex (M/F)	Sample size (E/C)	Age (years) (mean ± SD)	Outcome	Adverse events
Zhu et al. (7)	iTBS	Ipsilesional cerebellum	8/28	56.94 ± 47.23 (days)	27/9	18/18	60.5 ± 8.15	FMA-LE, BBS, TUG, BI	No
Xie et al. (3)	iTBS	Contralesional cerebellum	20/16	NA	24/12	18/18	53.38 ± 7.81	FMA-LE, TUG	No
Wang et al. (24)	LF-rTMS	Contralesional motor area	35/17	14.32 ± 5.82 (days)	19/33	27/25	61.34 ± 4.55	FMA-LE, BBS, BI	No
Qurat-ul-ain et a. (23)	A-tDCS	Ipsilesional motor area, cerebellum	44/22	14.72 ± 10.22	52/14	22/22/22	57.57 ± 5.58	TUG, BBS	Both sham and real tDCS groups reported mild adverse events including headache, tingling, itching, and skin redness
Choa et al. (41)	rTMS + tDCS	HF-rTMS on ipsilesional motor area tDCS on contralesional motor area	5/25	13.7 ± 5.62 (days)	17/13	15/15	59.43 ± 10.91	FMA-LE	No
Duan et al. (31)	C-tDCS	Contralesional motor area	91/0	NA	41/50	46/45	66.20 ± 9.53	FMA-LE, TUG	NA
Tahtis et al. (38)	dual-tDCS	The anode on the ipsilesional leg motor area The cathode on the contralesional leg motor area	14/0	22.5 ± 8.70 (days)	11/3	7/7	61.85 ± 12.89	TUG	No
Klomjai et al. (16)	dual-tDCS	The anode on the ipsilesional motor area The cathode on the contralesional motor area	19/0	3.5 ± 2.36 (months)	14/5	NA	57.2 ± 2.8	TUG	Both sham and real tDCS groups reported mild adverse events including cutaneous sensations, tingling, and mild headache
Toktas e al. (33)	A-tDCS	Ipsilesional motor area	NA	7.47 ± 4.34 (months)	NA	14/14	60.68 ± 9.42	FMA-LE, BBS, TUG	NA
Guan et al. (24)	HF-rTMS	Ipsilesional motor area	42/0	4.3 ± 3.75 (months)	30/12	21/21	58.55 ± 10.93	FMA-LE, BI	NA
Prathum et al. (39)	dual tDCS	A-tDCS on the ipsilesional motor area C-tDCS on the contralesional motor area	24/0	15.92 ± 2.94 (days)	16/8	12/12	57.75 ± 3.68	FMA-LE, TUG	Both sham and real tDCS groups reported mild adverse events including tingling, itching, burning sensation, and headache
Wang et al. (18)	LF-rTMS, HF-rTMS	LF-rTMS on the contralesional motor area HF-rTMS on the ipsilesional motor area	240/0	21.33 ± 3.07 (days)	157/83	80/80/80	63.96 ± 9.89	FMA-LE, BBS, BI	NA
Li et al. (8)	LF-rTMS, cTBS, bil-rTMS	LF-rTMS on the contralesional motor area cTBS on the right cerebellar hemisphere	71/19	3.7 ± 1.78 (months)	57/23	30/30/30	56.5 ± 7.95	BI	NA
Gong et al. (9)	LF-rTMS, rTMS + tDCS	LF-rTMS on the contralesional motor area ctDCS on the contralesional motor area	52/18	16.49 ± 5.55 (days)	44/16	15/15/15/15	62.11 ± 13.16	FMA-LE, BI	No
Lin et al. (19)	LF-rTMS	Contralesional motor area	22/10	37.05 ± 26.40 (days)	21/11	16/16	60.3 ± 11.26	FMA-LE, BI	One patient reported dizziness, one patient reported tingling and scalp pain
Yu et al. (25)	HF-rTMS	Left dorsolateral prefrontal cortex	10/8	1.18 ± 0.33 (months)	15/3	9/9	55.99 ± 12.03	FMA-LE, BBS, TUG	NA
Manjia et al. (34)	A-tDCS	Supplementary motor area	17/13	142.1 ± 42.90 (days)	17/13	15/15	62.95 ± 10.40	FMA-LE, TUG	NA
Sharma et al. (20)	LF-rTMS	Contralesional motor area	96/0	NA	67/29	47/49	53.85 ± 14.17	FMA-LE, BI	One participant in the real TMS group reported seizure
Chang et al. (35)	A-tDCS	Tibialis anterior area of the ipsilesional precentral gyrus	24/0	16.3 ± 5.6 (days)	NA	12/12	62.85 ± 10.61	FMA-LE, BBS	NA
Aneksan et al. (40)	dual-tDCS	The anode on the ipsilesional motor area The cathode on the contralesional motor area	25/0	95.52 ± 45.13 (days)	17/8	13/12	54.36 ± 12.35	TUG	Both sham and real tDCS groups reported mild adverse events including tingling sensation, skin redness, and headache
Wanga et al. (26)	HF-rTMS	Tibialis anterior area of the ipsilesional precentral gyrus	6/8	29.01 ± 20.4 (months)	11/3	8/6	54.01 ± 12.60	FMA-LE	No
Ling et al. (28)	iTBS	Ipsilesional motor area, contralesional cerebellum	12/24	59.28 ± 48.42 (days)	26/10	12/12/12	57.5 ± 12.25	FMA-LE, BBS, BI	Real iTBS group reported mild adverse events including headache and mild vertigo
Huang et al. (21)	LF-rTMS	Contralesional motor area	25/13	28.45 ± 21.78 (days)	23/15	18/20	61.67 ± 9.76	FMA-LE, TUG, BI	NA
Wang et al. (22)	LF-rTMS	Contralesional motor area	NA	1.92 ± 1.17 (years)	15/9	12/12	63.94 ± 11.43	FMA-LE	No
Lin et al. (29)	iTBS	Bilateral motor area	16/4	371.5 ± 220.33 (days)	17/13	10/10	60.95 ± 8.70	FMA-LE, BBS, TUG, BI	NA
Bornheim et al. (36)	A-tDCS	Ipsilesional motor area	50/0	NA	33/17	25/25	62.98 ± 12.29	FMA-LE, BI	Both sham and real tDCS groups reported mild adverse events including a slight tingling, itching, burning sensation, and slight headache
Madhavan et al. (37)	A-tDCS	Ipsilesional motor area	18/12	5.16 ± 3.95 (years)	14/16	19/21	58 ± 10.40	FMA-LE, BBS, TUG	No
Koch et al. (30)	iTBS	Cerebellar	34/0	13.09 ± 17.19 (months)	23/11	17/17	64 ± 11.39	BBS, BI	No
Rastgoo et al. (23)	LF-rTMS	Ipsilesional motor area	15/5	28.8 ± 18.76	16/4	10/10	52.15 ± 11.36	FMA-LE, TUG	No

### 3.3 Quality assessment

**Figure 2:**
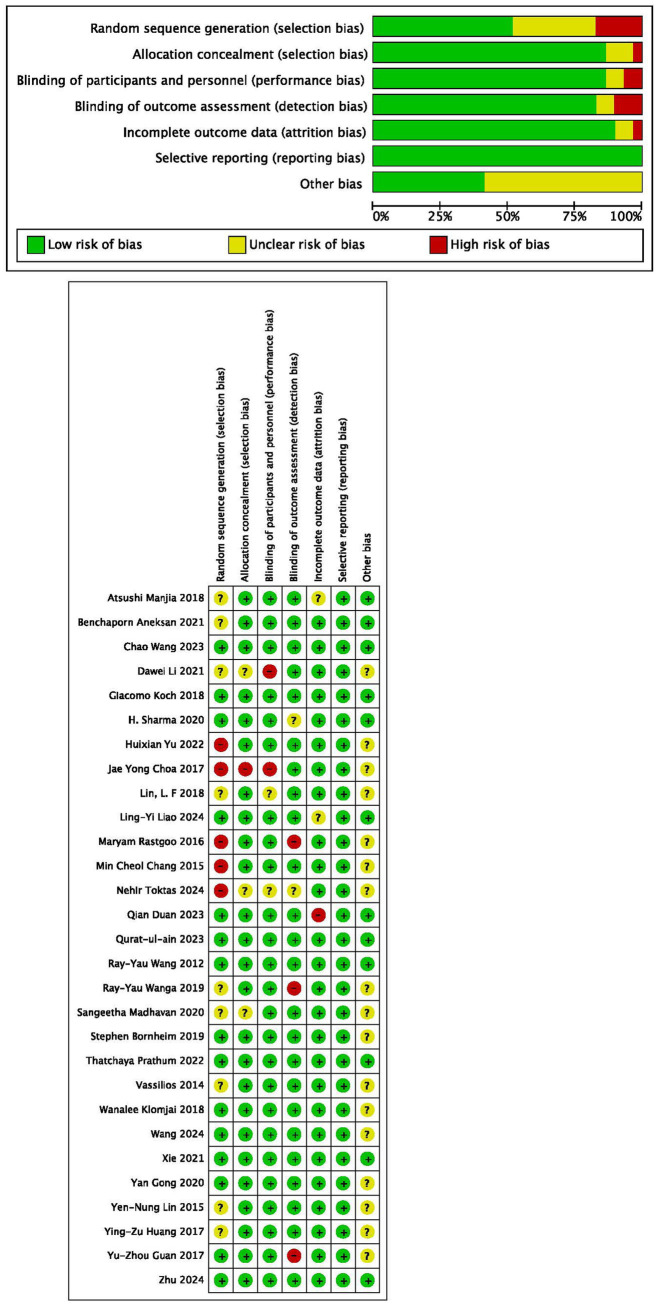
Assessment of the risk of bias in the included studies.

Among all the 29 selected studies included, 52% reported random sequence generation, 86% reported allocation concealment, 86% implemented blinding of participants and personnel, 83% implemented blinding of outcome assessment, and 90% provided incomplete outcome data (Figures 2A,B). Egger’s test results for different outcomes—FMA-LE (*p* = 0.586), TUG (*p* = 0.072), BBS (*p* = 0.542), and MBI (*p* = 0.298)—suggested a lack of evidence of publication bias.

### 3.4 Network geometry of interventions

**Figure 3:**
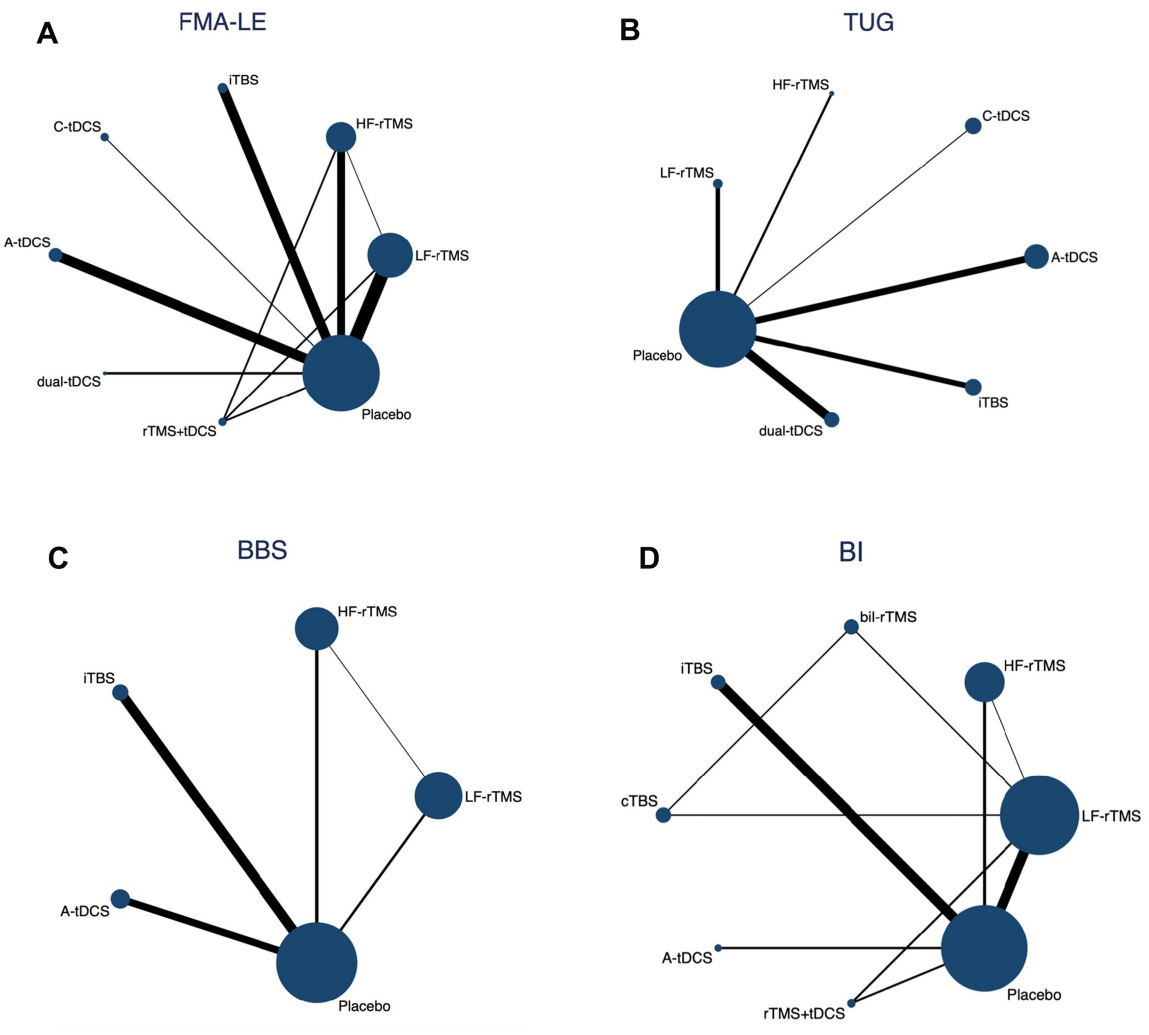
Network geometry of different outcome measures. Nodes are connected by a line when treatments are directly comparable. The width of each line is proportional to the number of randomized controlled trials, and the size of each node is proportional to the number of patients (sample size).

A network graph illustrating different NiBS treatments for improving lower extremity motor function is presented in Figure 3.

### 3.5 Synthesis of results

#### 3.5.1 FMA-LE

**Figure 4:**
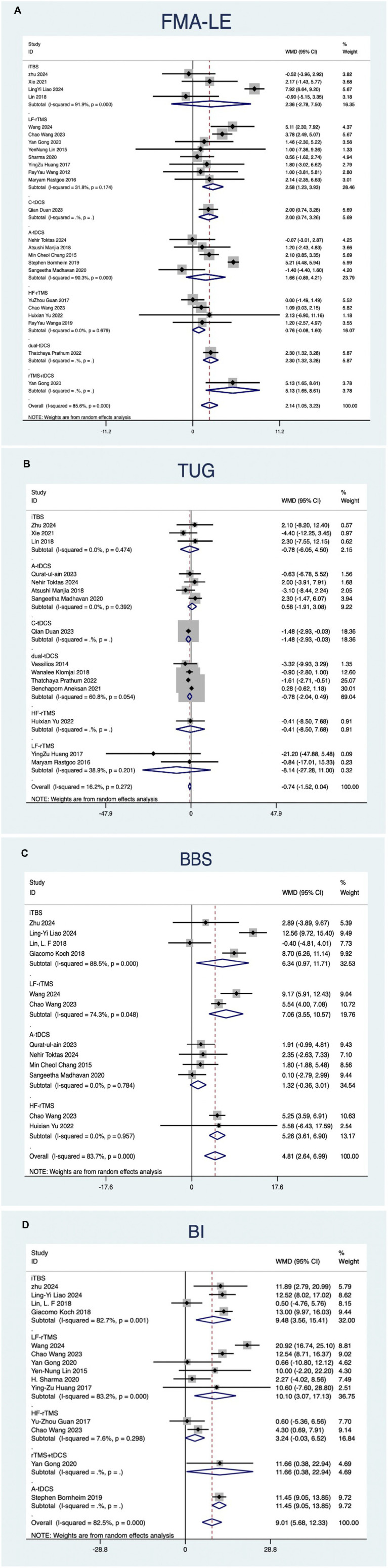
Forest plots of network meta-analyses for different outcome measures compared with placebo.

The NMA of NiBS treatments for lower extremity motor recovery, using FMA-LE as the outcome measure, included 23 studies. Pairwise meta-analysis suggested that LF-rTMS (MD, 2.58; 95% CI, 1.23 to 3.93), C-tDCS (MD, 2.00; 95% CI, 0.74 to 3.26), and dual-tDCS (MD, 2.30; 95% CI, 1.32 to 3.28) were significantly more effective than placebo (Figure 4A).

**Figure 5:**
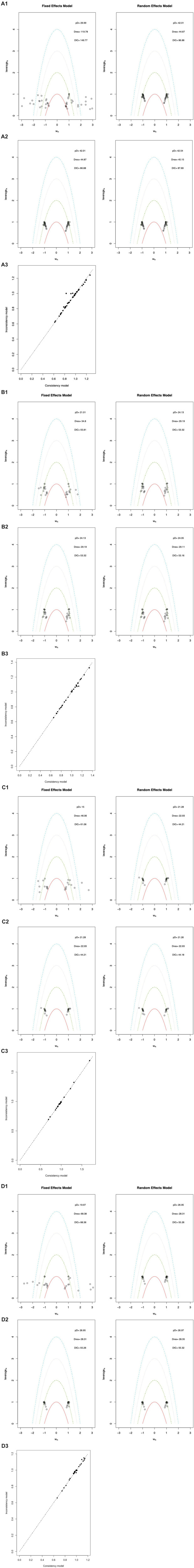
Leverage plots and fit statistics for different outcome measures. DIC, deviance information criterion.

**Figure 6:**
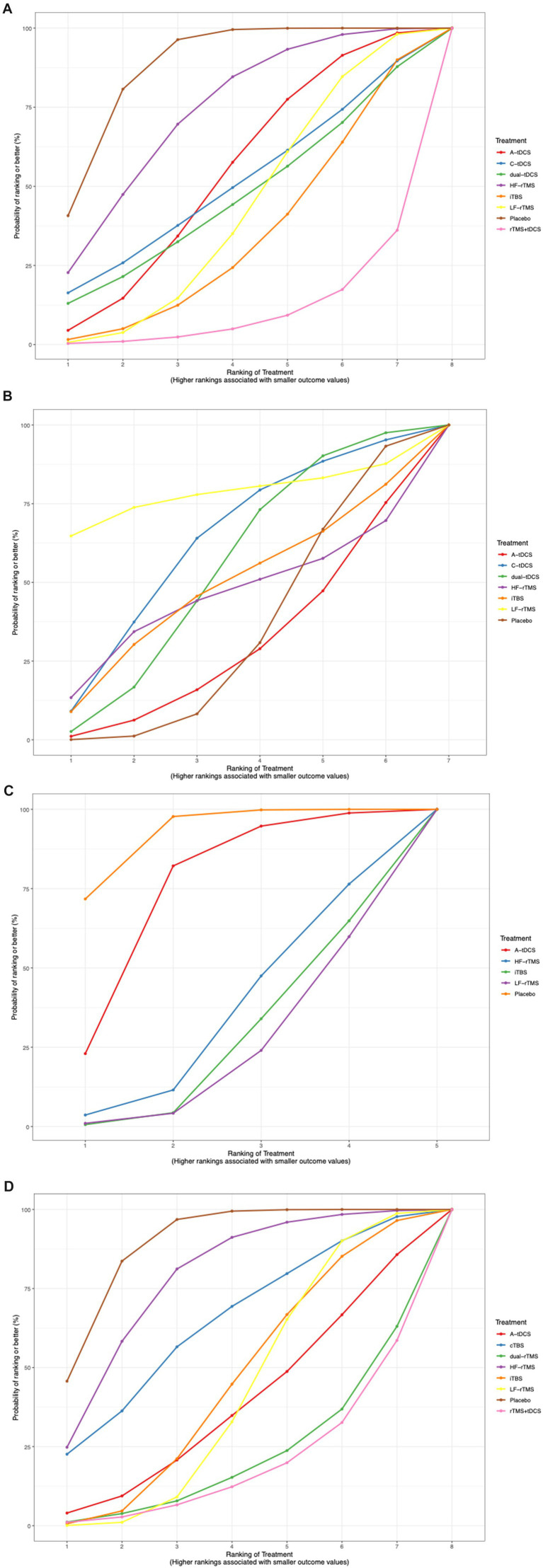
League table summarizing the results of the indirect comparisons of different outcome measures. Numbers in the cells denote the mean incidence risk rate (95% confidence interval). ** **p-value < 0.05.

Regarding the NMA results, we compared the DIC of the fixed and random models. The DIC of the random model was lower than that of the fixed model (86.88 vs. 149.77) (Figure 5A1). We chose to use the random model for the NMA. The results indicated that LF-rTMS (MD, 2.36; 95% CI, 0.16 to 4.49) and rTMS + tDCS (MD, 5.26; 95% CI, 0.96 to 9.50) were significantly more effective than placebo (Figure 6A).

**Figure 7:**
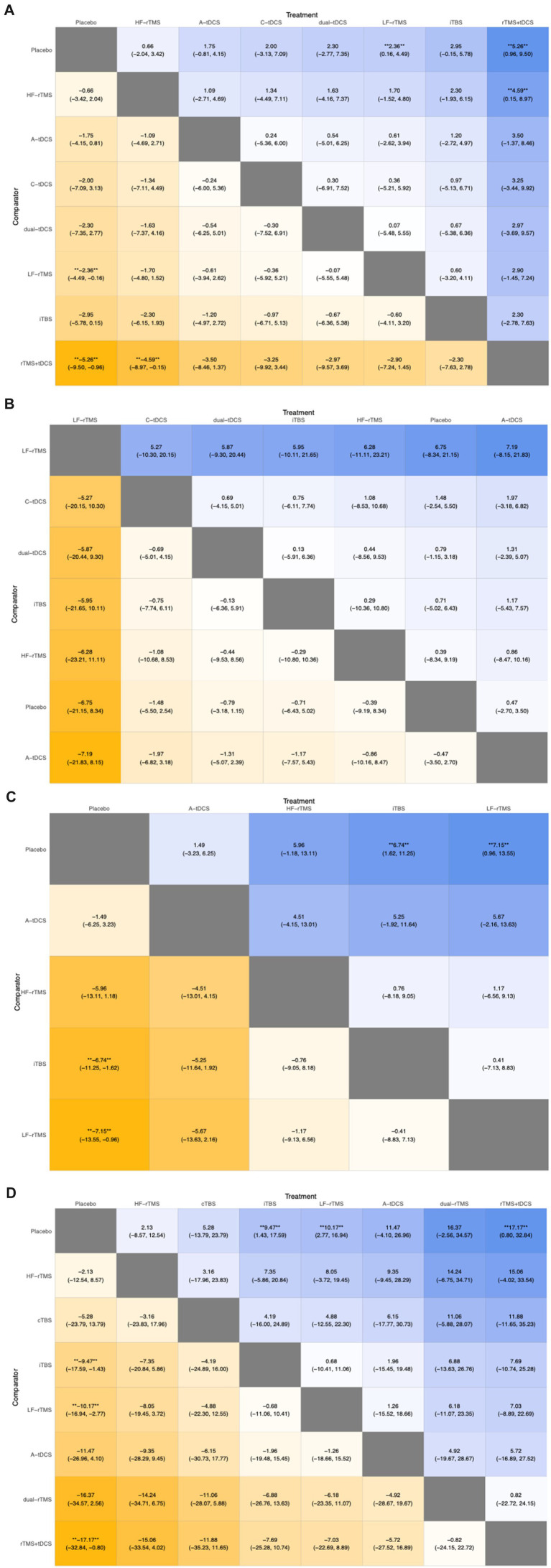
Rankings of the effects of different outcomes shown with SUCRAs.

The SUCRA plot ranked rTMS + tDCS as the most effective treatment for improving lower extremity motor function after stroke, followed by LF-rTMS, iTBS, A-tDCS, dual-tDCS, C-tDCS, and HF-rTMS (Figure 7A).

#### 3.5.2 TUG

The NMA of NiBS treatments for improving walking function, using the TUG test as the outcome, included 15 studies. Pairwise meta-analysis suggested that no NiBS treatment was significantly more effective than placebo (Figure 4B).

For the NMA results, we compared the DIC of the fixed and random models. The DIC of the random model was lower than that of the fixed model (53.32 vs. 55.81) (Figure 5B1). We used the random model for the NMA. Results from the NMA suggested that no NiBS treatment was significantly more effective than placebo (Figure 6B).

The SUCRA plot indicated that LF-rTMS ranked highest for improving walking function in stroke, followed by HF-rTMS, C-tDCS, iTBS, dual-tDCS, and A-tDCS (Figure 7B).

#### 3.5.3 BBS

The NMA of NiBS treatments for enhancing body balance function, using the BBS as the outcome, included 11 studies. Pairwise meta-analysis indicated that iTBS (MD, 6.34; 95% CI, 0.97 to 11.71), LF-rTMS (MD, 7.06; 95% CI, 3.55 to 10.57), and HF-rTMS (MD, 5.26; 95% CI, 3.61 to 6.90) were significantly more effective than placebo (Figure 4C).

For the NMA results, we compared the DIC of the fixed and random models. The DIC of the random model was lower than that of the fixed model (44.21 vs. 61.06) (Figure 5C1). We used the random model for the NMA. Results from the NMA showed that iTBS (MD, 6.74; 95% CI, 1.62 to 11.25) and LF-rTMS (MD, 7.15; 95% CI, 0.96 to 13.55) were significantly more effective than placebo (Figure 6C).

The SUCRA plot suggested that iTBS was the highest-ranked treatment for improving body balance function in stroke, followed by LF-rTMS, HF-rTMS, and A-tDCS (Figure 7C).

#### 3.5.4 BI

The NMA of NiBS treatments for improving activities of daily living, using the BI as the outcome, included 13 studies. Pairwise meta-analysis showed that iTBS (MD, 9.48; 95% CI, 3.56 to 15.41), A-tDCS (MD, 11.45; 95% CI, 9.05 to 13.85), rTMS + tDCS (MD, 11.66; 95% CI, 0.38 to 22.94), and LF-rTMS (MD, 10.10; 95% CI, 3.07 to 17.13) were significantly more effective than placebo (Figure 4D).

For the NMA results, we compared the DIC values of the fixed and random models. The DIC of the random model was lower than that of the fixed model (55.26 vs. 88.36) (Figure 5D1). We selected the random model for the NMA. Results from the NMA indicated that iTBS (MD, 9.47; 95% CI, 1.43 to 17.59), LF-rTMS (MD, 10.17; 95% CI, 2.77 to 16.94), and rTMS + tDCS (MD, 17.17; 95% CI, 0.80 to 32.84) were significantly more effective than placebo (Figure 6D).

The SUCRA plot indicated that LF-rTMS was the most effective treatment for enhancing activities of daily living in stroke patients, followed by iTBS, rTMS + tDCS, dual-rTMS, A-tDCS, cTBS, and HF-rTMS (Figure 7D).

### 3.6 Assessment of statistical inconsistency

To evaluate global-level consistency, we compared the DIC between the consistency and inconsistency models. The results indicated that the difference in DIC was less than 5, with the consistency model showing a lower DIC than the inconsistency model across all selected outcomes (Figure 5). For local inconsistency, the leverage plots demonstrated that the data points were distributed along the slanting stitch, suggesting no evidence of inconsistency within any loop. Overall, the statistical assessment revealed no indication of inconsistency within the network.

### 3.7 Adverse effects

Only one case of seizure occurred after rTMS (20). No severe adverse events related to NiBS were reported in any of the included studies. Some studies reported mild adverse reactions, such as headaches, burning sensations, slight tingling, and itching, which resolved quickly after treatment and caused no long-term effects.

## 4 Discussion

To the best of our knowledge, this study represents the first NMA to examine the effectiveness of NiBS on poststroke lower extremity motor function. The analysis evaluated the efficacy of nine different NiBS treatments compared with placebo in 1319 participants with poststroke lower extremity disorders. For the primary outcome, measured using the FMA-LE, the NMA found that LF-rTMS and rTMS + tDCS were more effective than placebo. Pairwise meta-analysis also indicated that LF-rTMS, C-tDCS, and dual-tDCS were significantly more effective than placebo. Regarding walking function, assessed by the TUG test, both direct and indirect evidence showed that no NiBS intervention was more effective than placebo. The NMA assessment of body balance function revealed that iTBS and LF-rTMS were more effective than placebo. Pairwise meta-analysis suggested that iTBS, LF-rTMS, and HF-rTMS exceeded placebo in effectiveness. For activities of daily living, evaluated using the BI, direct evidence indicated that iTBS, A-tDCS, rTMS + tDCS, and LF-rTMS were more effective than placebo. The NMA results for BI demonstrated that iTBS, LF-rTMS, and rTMS + tDCS outperformed placebo.

The main stimulation modes of TMS included in this study were LF-rTMS and iTBS. For the recovery of hand motor function during the subacute phase of stroke, existing evidence and definite efficacy suggest a level A recommendation for LF-rTMS (42). A meta-analysis confirmed the therapeutic effect of LF-rTMS on lower limb movement disorders after stroke (3). Our research demonstrated that the effect of LF-rTMS on motor function recovery, body balance, and activities of daily living was superior to that of placebo in poststroke patients. iTBS, a novel TMS mode that functions in the opposite way of LF-rTMS, enhances nervous system excitability. iTBS should be considered a level B recommendation for treating lower-limb spasticity 字段 (42). Our investigation suggests that iTBS could improve activities of daily living and body balance in poststroke patients.

Regarding tDCS, previous meta-analyses and our own research have demonstrated its restorative effects in poststroke patients (11, 43). However, the number of RCTs assessing each effective tDCS mode was relatively small in this systematic review. Similarly, in the NMA of the primary outcome, although rTMS + tDCS appeared to be the most effective stimulation method, only two relevant RCTs were included (9, 41). Additional clinical studies are needed to evaluate the effects of tDCS in addressing lower extremity dysfunction after stroke.

To date, NiBS treatments for poststroke motor dysfunction mainly follow the interhemispheric inhibition model. This model indicates that the two hemispheres suppress each other’s excitability via nerve fiber bundles in the corpus callosum, maintaining a dynamic balance. After a stroke, the inhibitory effect of the affected hemisphere diminishes, disrupting this balance. The unaffected hemisphere then suppresses the excitability of the affected hemisphere through the corpus callosum, causing a decline in motor function (44). Nervous system excitability is affected by synaptic connections and efficacy, which NiBS modulates through mechanisms tied to long-term potentiation or depression (45, 46). To enhance poststroke limb dysfunction, inhibitory stimulation should be applied to the contralesional motor area (17, 31), whereas excitatory NiBS stimulation should focus on the ipsilesional motor area (4, 26, 36). Adhering to the interhemispheric inhibition model (HF-rTMS on the ipsilesional motor cortex and LF-rTMS on the contralesional side), one study investigated how rTMS influences motor function and cortical activation. Compared to the sham group, the real rTMS group exhibited motor improvements. fMRI data indicated a link between motor gains and increased cortical excitability caused by rTMS (47). Another study showed that applying A-tDCS to the primary motor cortex of stroke patients increased connectivity within the EEG network of the ipsilesional motor cortex. This heightened connectivity was linked to greater corticospinal excitability after A-tDCS (48). Notably, our NMA included a rare study exploring the effects of rTMS on the left dorsolateral prefrontal cortex (25), a region more commonly targeted to enhance cognitive function or treat depression (49). For poststroke motor dysfunction, the dorsolateral prefrontal cortex was rarely used as a stimulation target. Some included studies explored the improvement of poststroke lower limb dysfunction by using NiBS on the cerebellum (7, 27, 28, 30, 32). A study demonstrated that, compared to sham stimulation, cerebellar iTBS enhanced post-stroke body balance and lower limb function, along with an increase in motor-evoked potential amplitudes (28) regulatory center for movement. During exercise, the cerebellum receives and integrates information from the cerebral cortex, muscles, and joints. Based on this mechanism, the cerebellum presents a feasible target for modulating motor behavior and treating motor impairments caused by stroke (50). A study investigating poststroke dysphagia suggested that bilateral cerebellar iTBS can effectively enhance swallowing function (51). In treating post-stroke upper limb spasticity, cerebellar iTBS enhances the effects of conventional physical therapy (52). In a healthy population, another study found that active cerebellar rTMS restores swallowing accuracy and inhibitory effects caused by a cortical “virtual lesion” on pharyngeal motor-evoked potentials (53). In speech improvement, right cerebellar tDCS was found to significantly enhance phonemic fluency. This improvement is also linked to increased functional connectivity (54). Based on these promising findings, the cerebellum could be a crucial target for NiBS interventions in poststroke motor rehabilitation. However, more research is needed to develop a standardized approach to translate small-scale experimental results into a wide range of clinical practices (55).

Our investigation reported only one case of a severe adverse reaction (seizure) related to rTMS (20), Although causality between the seizure and rTMS treatment was not confirmed, numerous mild adverse events have been reported. These mainly involve skin sensations, are short in duration, and have no sequelae. According to the published TMS safety guidelines (56), seizure induction is the most severe acute adverse event; however, the risk of rTMS-induced seizures is definitely low. A review that included 41 reports published up to February 2020 examined TMS-induced seizures (57). Among these 41 reports, 13 involved healthy individuals, and 28 involved patients. Due to the inconsistent distribution of TMS patterns among the reports (19 HF-rTMS, 1 LF-rTMS, 8 single-pulse TMS, 9 deep TMS, 2 iTBS, 1 cTBS, and 1 unknown), it was difficult to identify a correlation between TMS-induced seizure and specific populations or TMS patterns. Regarding tDCS, our review found no severe adverse events and only mild adverse events similar to those of rTMS, with short duration and no sequelae. Previous safety guidelines have confirmed the safety of tDCS (58). However, given the widespread use of home-based tDCS devices (39), untrained application may cause burns, reduced accuracy, and other complications. Professional guidance is necessary before use. Theoretically, the combination of rTMS and tDCS could raise the incidence of severe adverse events (59); however, our review did not report any such cases (9, 41). Similarly, a study involving patients with depression reported no serious adverse events, except for increased scalp pain when rTMS was applied before tDCS (60). In a healthy population, another review found no serious adverse events related to combined interventions (61). In brief, there is no current evidence questioning the safety of the combination of tDCS and rTMS.

This study has several limitations. First, the analysis using TUG as the outcome measure indicated that, compared with the placebo group, NiBS did not appear to improve patients’ walking function. This result may be due to the fact that, in some of the included clinical studies, the baseline walking function of the experimental group was weaker than that of the control group (7, 29, 33). Second, previous studies reported varying efficacies of NiBS depending on the stage of stroke (5). Although our review included patients at different stages of stroke onset, a subgroup analysis of NiBS treatment effects by stroke stage was not performed due to limited relevant research. Additionally, the NMA did not encompass all NiBS interventions, such as tRNS, taVNS, and tACS. There is a lack of suitable studies on these interventions for lower-extremity motor function (11, 62).

### 4.1 Conclusion

The meta-analysis suggests that LF-rTMS and rTMS + tDCS are effective neurostimulation therapies for enhancing poststroke lower limb motor function. Probability ranking indicated that, among all the NiBS interventions analyzed, rTMS + tDCS may be the most effective. Concerning body balance function, iTBS and LF-rTMS improved poststroke balance, with iTBS potentially being the most effective. For activities of daily living, iTBS, LF-rTMS, and rTMS + tDCS demonstrated beneficial effects, with LF-rTMS possibly being the most effective among them.

## Data Availability

The datasets presented in this study can be found in online repositories. The names of the repository/repositories and accession number(s) can be found in the article/Supplementary material.

## Footnotes

^1^ http://www.crd.york.ac.uk/prospero/
