# Supplementary material for: Non-invasive brain stimulation for the improvement of lower extremity motor function in patients with stroke: a systematic review and network meta-analysis
Source: Front Neurol. 2025 Dec 1;16:1664707. doi: 10.3389/fneur.2025.1664707 (PMC12702769; doi:10.3389/fneur.2025.1664707)
Supplement: Supplementary file 1 [file Table_1.docx]

**FMA-LE**


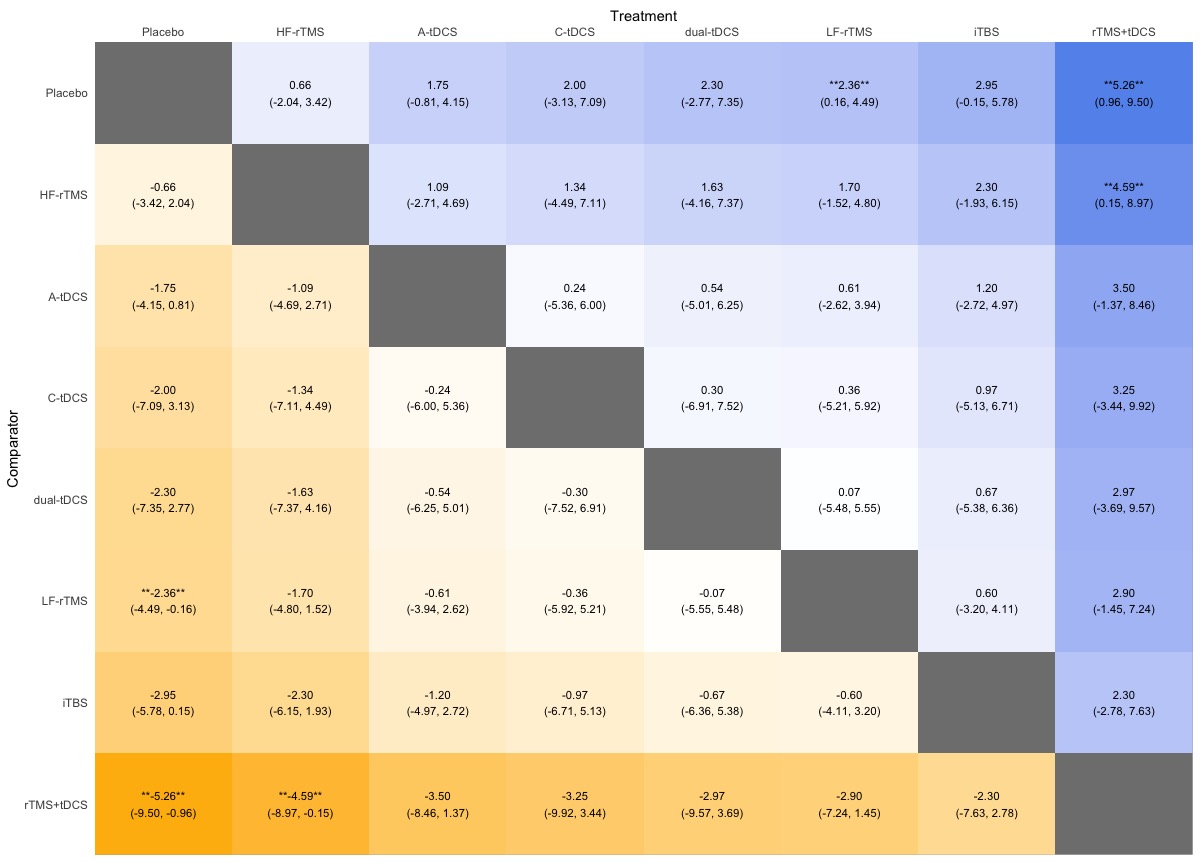


**TUG**


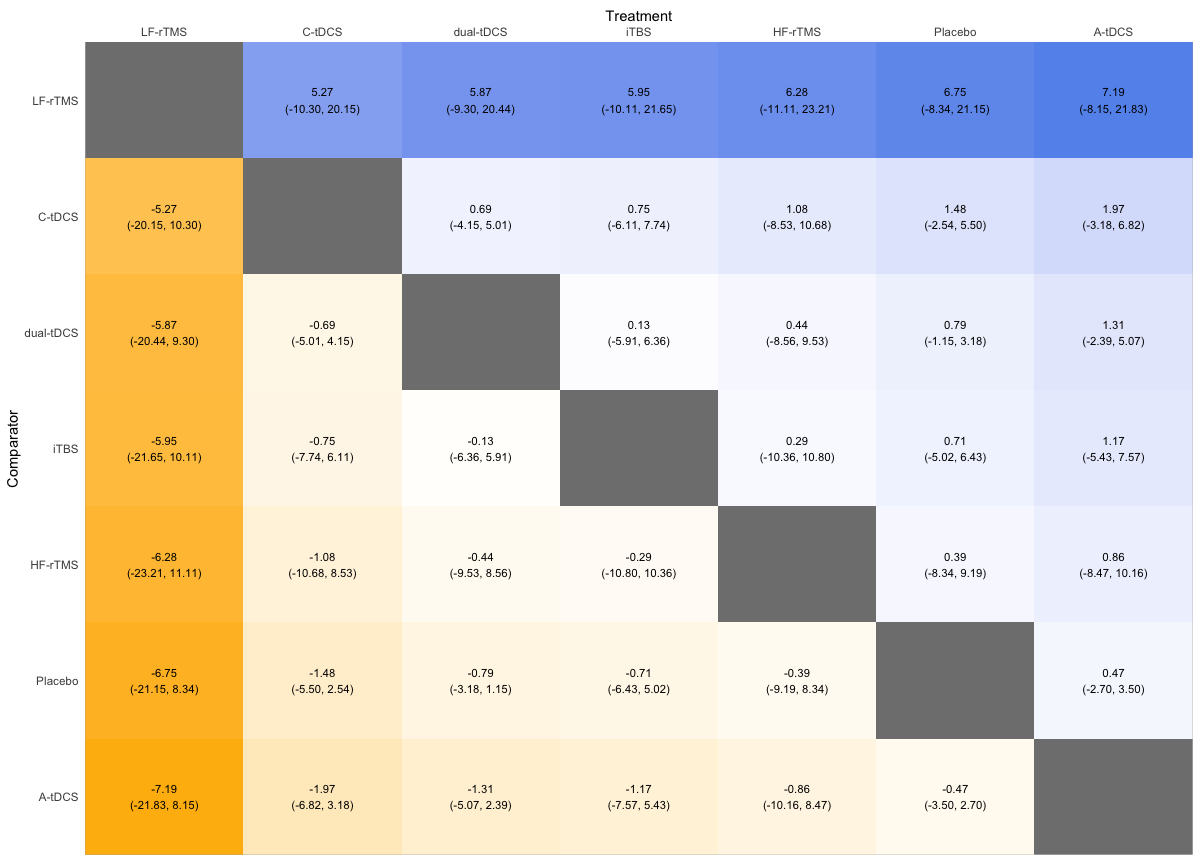


BI


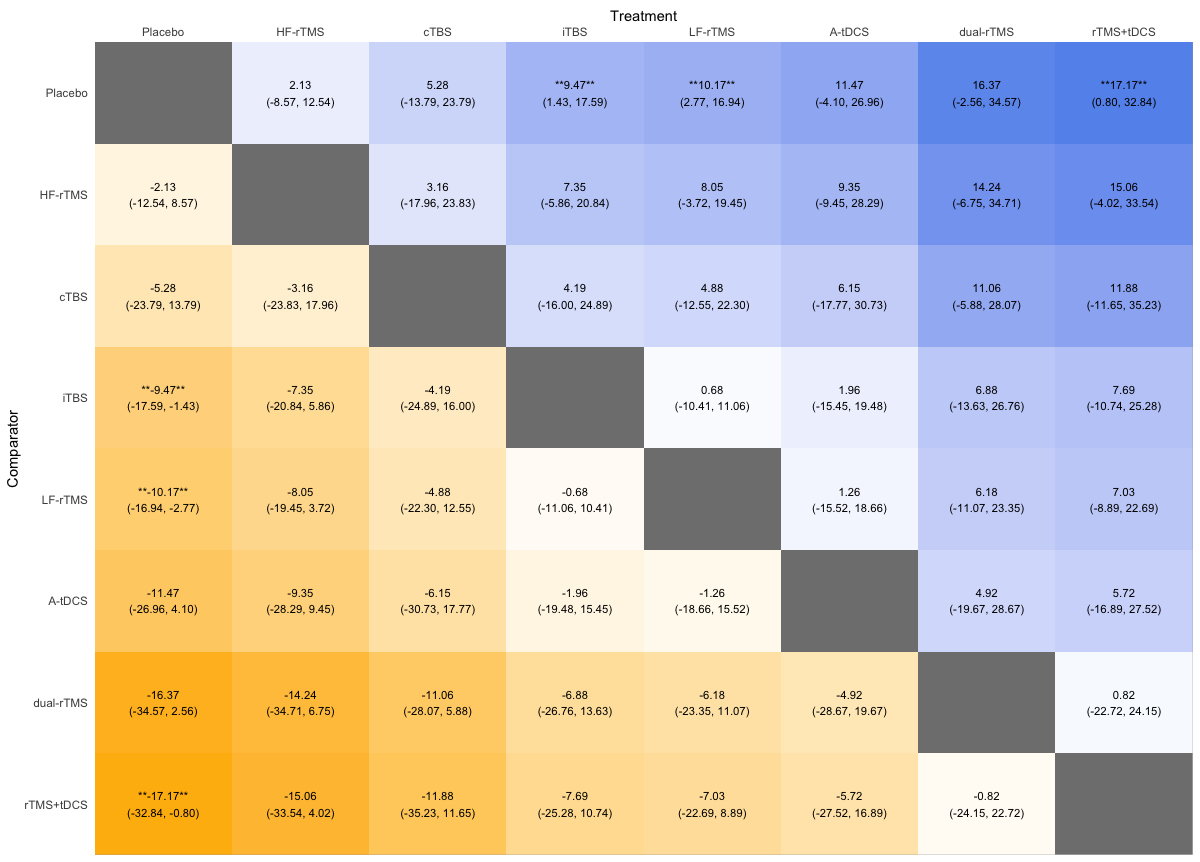


**BBS**


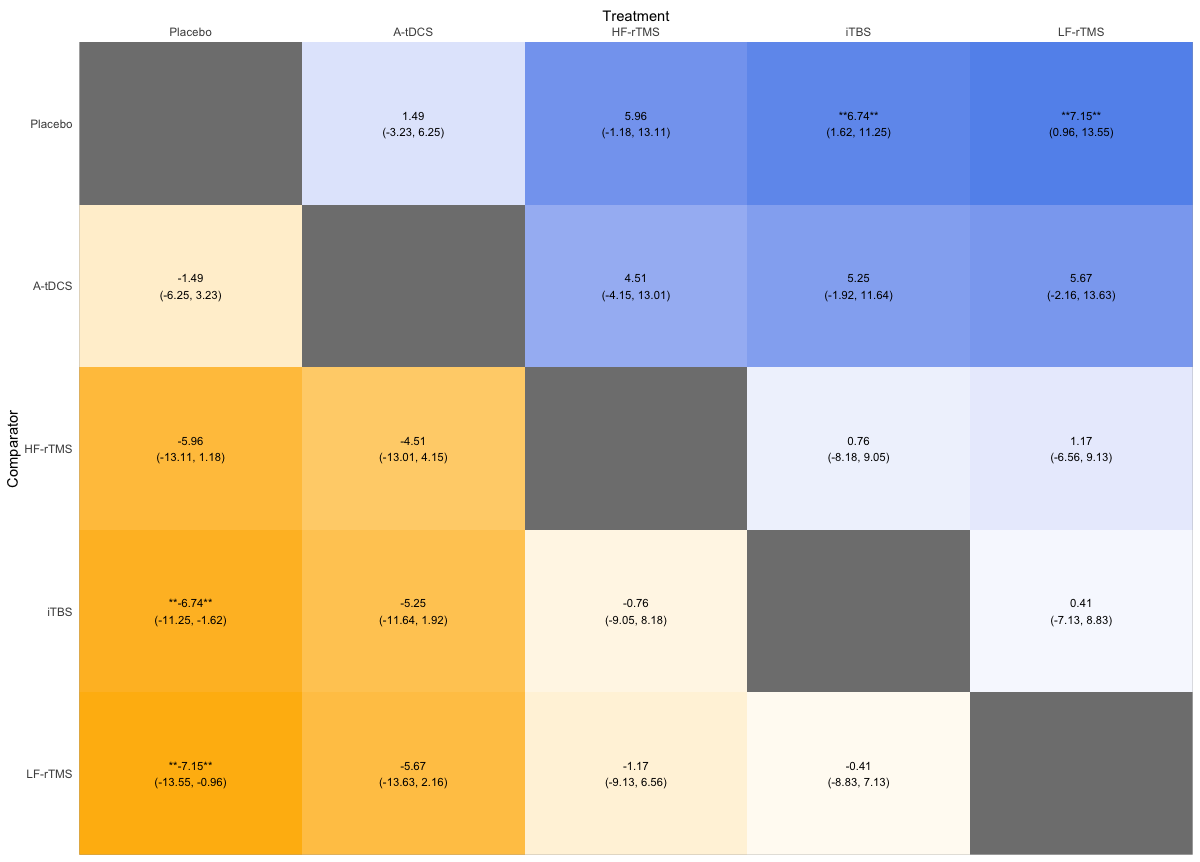


**Fig 7 League table summarizing the results of the indirect comparisons of different outcome measure**

The numbers in the cells denote the mean incidence risk rate (95 % confidence interval).

** **: P value < 0.05.
