# Supplementary figures and images for: Non-invasive brain stimulation for the improvement of lower extremity motor function in patients with stroke: a systematic review and network meta-analysis
Source: Front Neurol. 2025 Dec 1;16:1664707. doi: 10.3389/fneur.2025.1664707 (PMC12702769; doi:10.3389/fneur.2025.1664707)

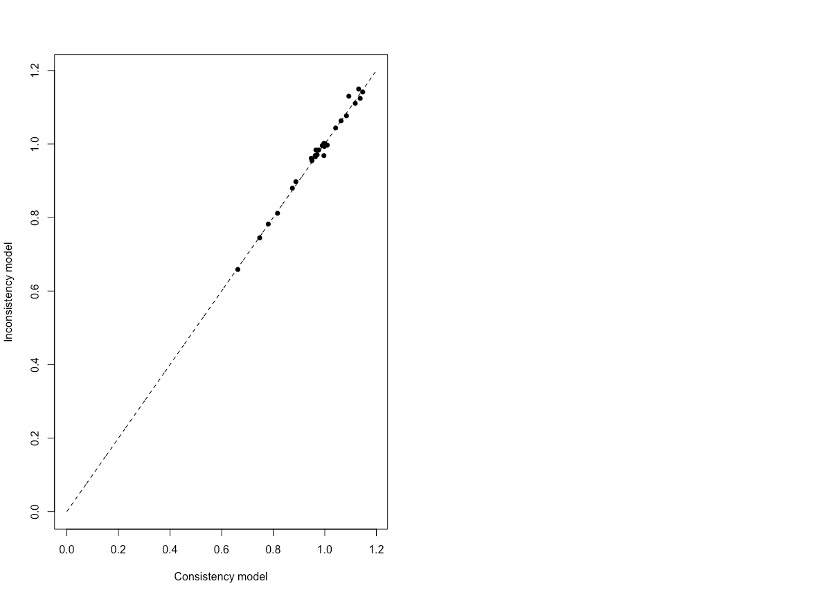

Supplement: Supplementary file 3 [file Image_1.jpeg]

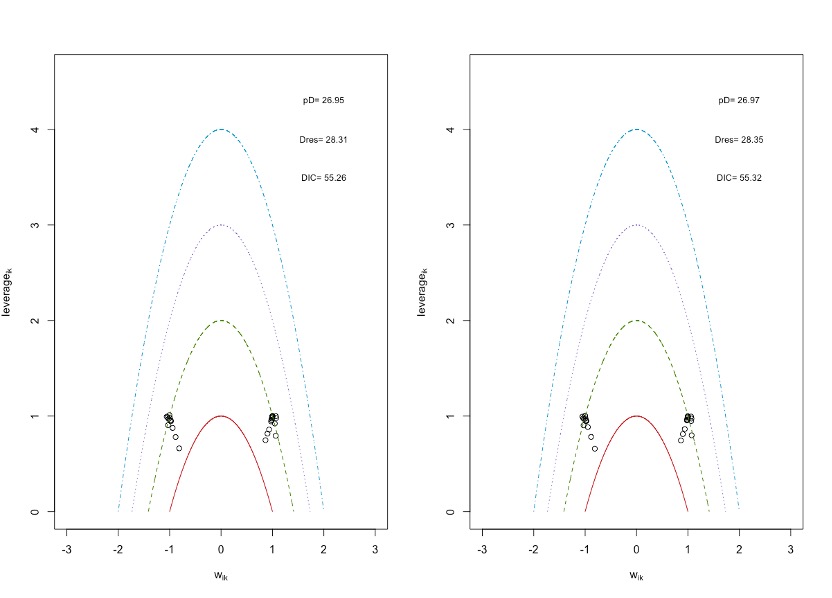

Supplement: Supplementary file 4 [file Image_2.jpeg]

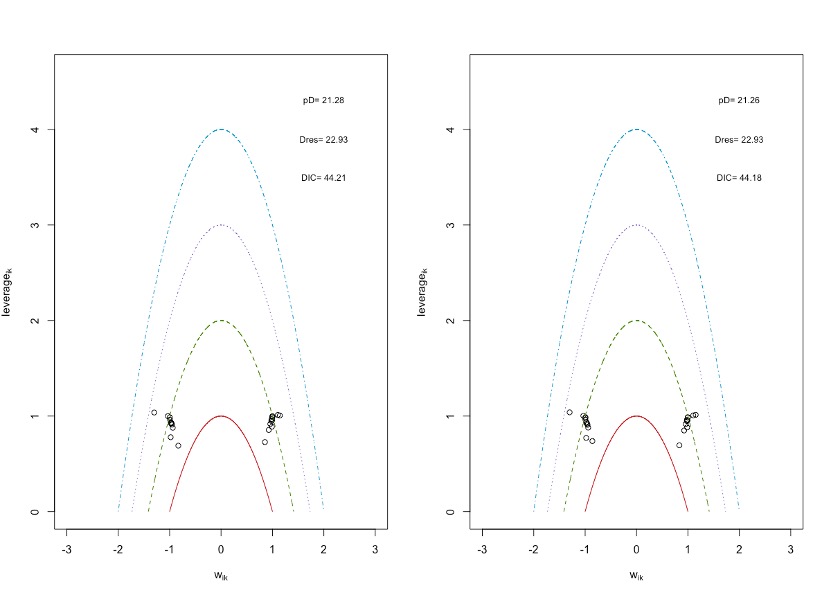

Supplement: Supplementary file 5 [file Image_3.jpeg]

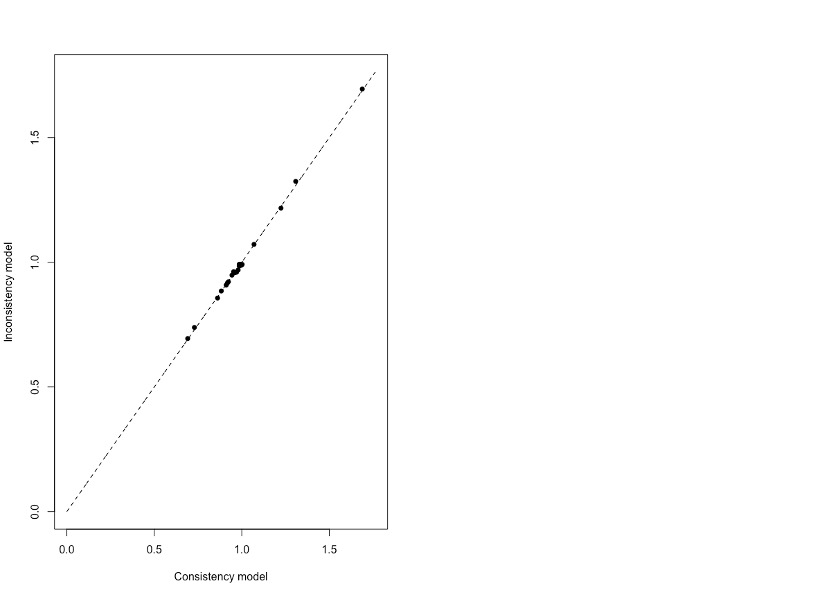

Supplement: Supplementary file 6 [file Image_4.jpeg]

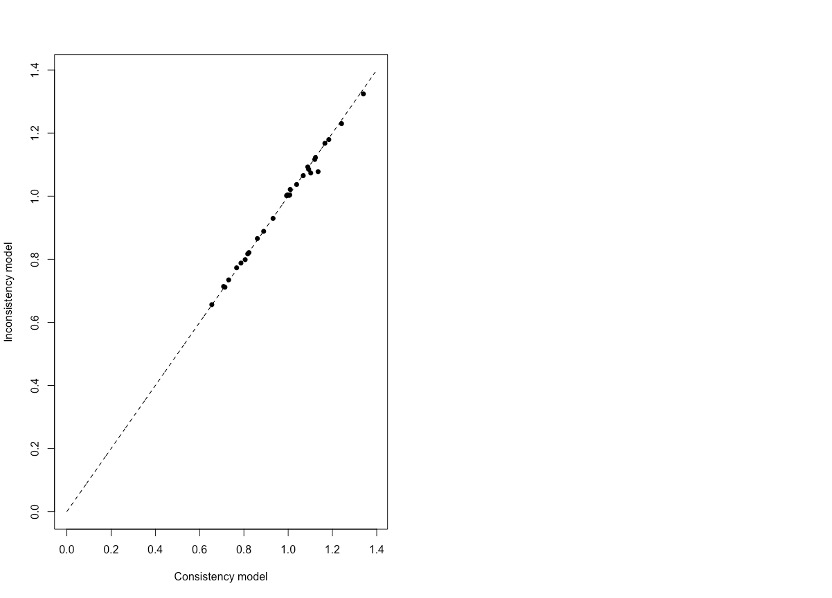

Supplement: Supplementary file 7 [file Image_5.jpeg]

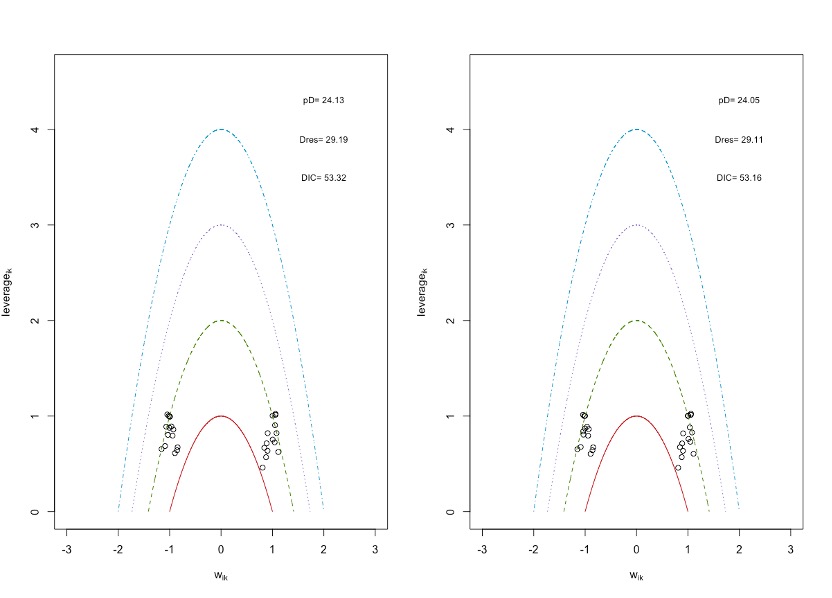

Supplement: Supplementary file 8 [file Image_6.jpeg]

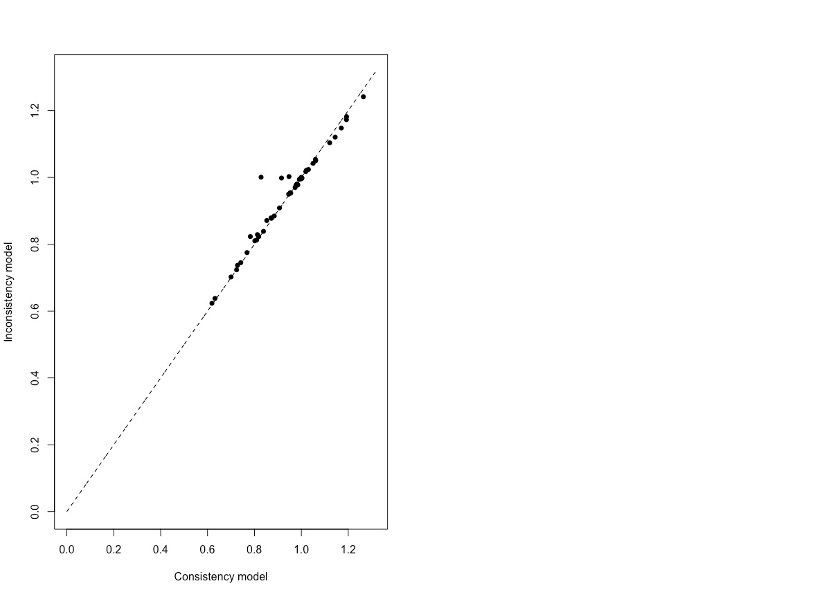

Supplement: Supplementary file 9 [file Image_7.jpeg]

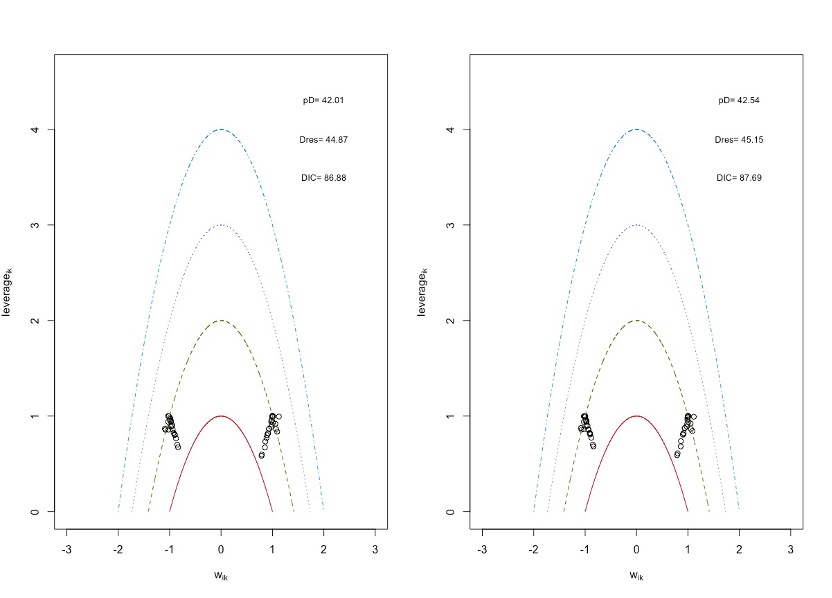

Supplement: Supplementary file 10 [file Image_8.jpeg]

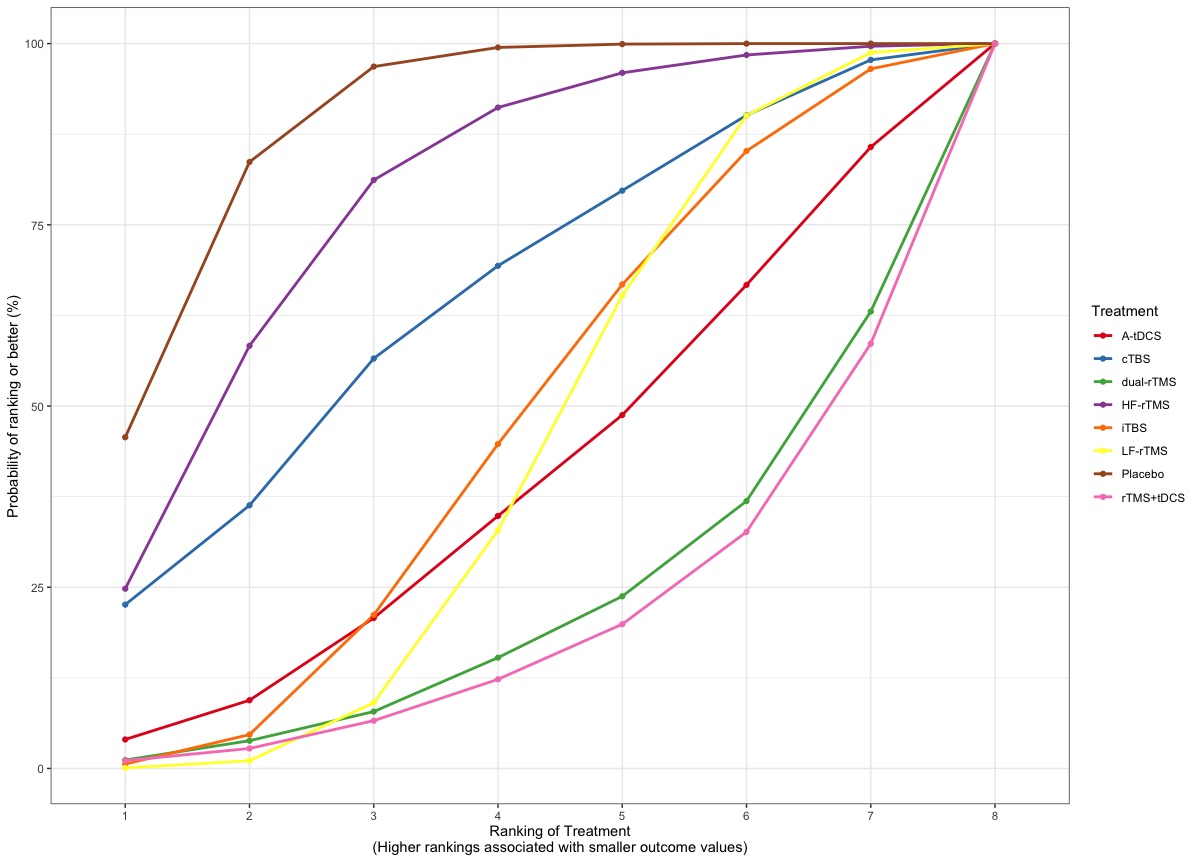

Supplement: Supplementary file 11 [file Image_9.jpeg]

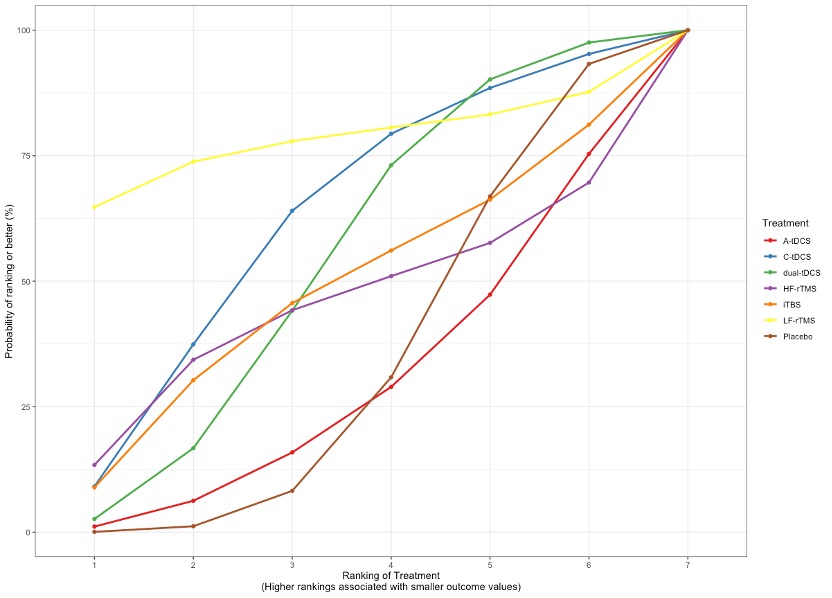

Supplement: Supplementary file 12 [file Image_10.jpeg]

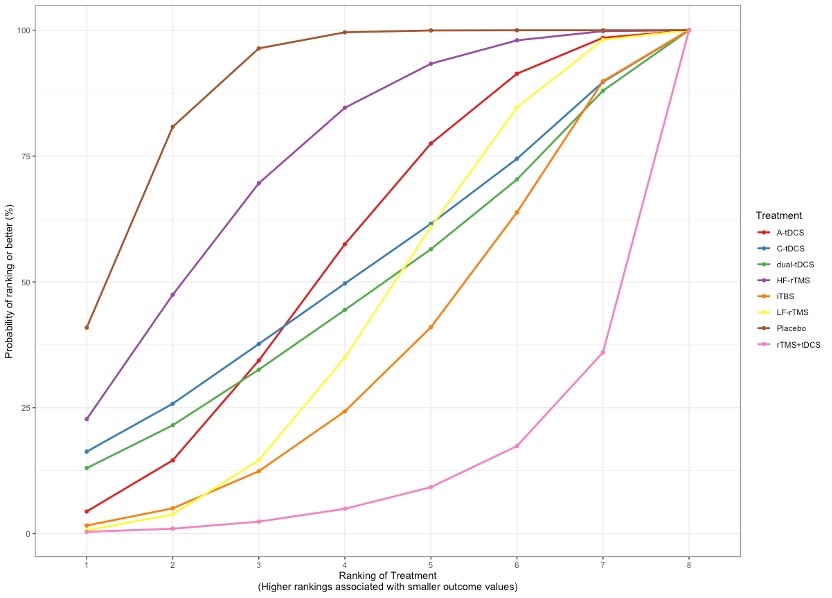

Supplement: Supplementary file 13 [file Image_11.jpeg]

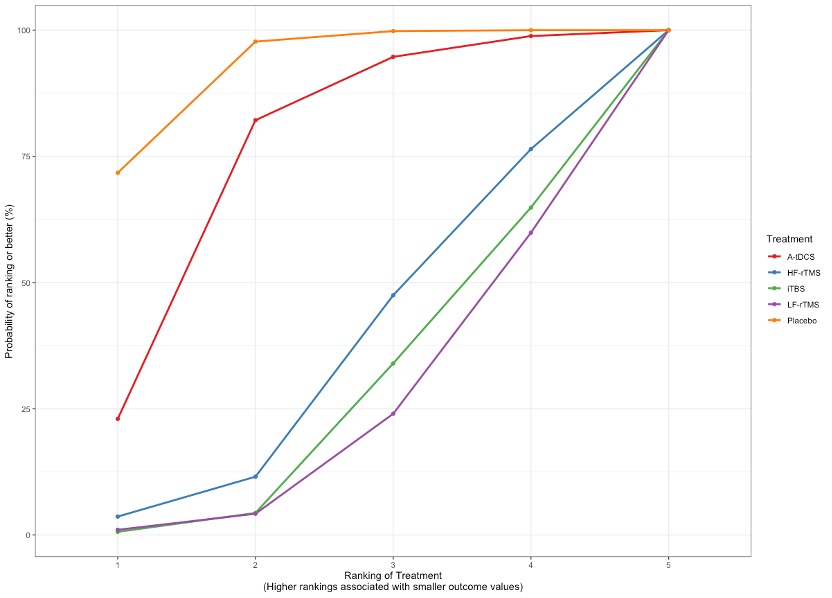

Supplement: Supplementary file 14 [file Image_12.jpeg]
